# Optimal nutritional support is key to liberation from mechanical ventilation

**DOI:** 10.62675/2965-2774.20260049

**Published:** 2026-03-16

**Authors:** Silvia De Rosa, Sergio Lassola

**Affiliations:** 1 University of Trento Center for Medical Sciences Trento Italy Center for Medical Sciences - CISMed, University of Trento - Trento, Italy.; 2 Santa Chiara Hospital Department of Anesthesia and Intensive Care Trento Italy Department of Anesthesia and Intensive Care, Santa Chiara Hospital - Trento, Italy.

**Keywords:** Respiration, artificial, Ventilator weaning, Critical illness, Respiratory muscles, Nutrients, Metabolic tolerance, Diaphragm dysfunction, Nutritional support, Energy metabolism, Vitamin D

## Abstract

Liberation from mechanical ventilation is a physiologically demanding phase of critical illness that requires coordinated recovery of respiratory muscle function, metabolic stability, and systemic resilience. Nutritional therapy plays a central role in supporting this transition. However, early aggressive caloric and protein delivery has not improved outcomes and may be detrimental. Current evidence supports a phase-adapted, individualized approach: permissive underfeeding during acute inflammation, moderate protein provision, monitoring of metabolic tolerance, and targeted correction of micronutrient deficiencies. Future research should incorporate time to successful weaning as a clinically meaningful endpoint when evaluating nutritional interventions in the intensive care unit.

## INTRODUCTION

Liberation from mechanical ventilation is not simply a respiratory decision but also a metabolic test. It represents a complex metabolic and functional turning point in critical illness. Transitioning from controlled ventilation to spontaneous breathing increases diaphragm workload, oxygen consumption, and systemic energy demand, while patients frequently exhibit muscle wasting, insulin resistance, and persistent inflammation.^(1)^ Despite decades of research in intensive care unit (ICU) nutrition, the optimal strategy during weaning remains incompletely defined.^(2)^ Importantly, the effectiveness of medical nutrition therapy is profoundly influenced by the underlying inflammatory and infectious burden. Ongoing systemic inflammation drives catabolism, accelerates muscle protein breakdown, and may blunt anabolic responses to nutrient delivery.^(1)^ In this setting, nutritional support must be aligned with the evolving metabolic phase rather than guided by fixed targets.

### Energy delivery: respecting the metabolic phase

During the acute phase of critical illness, endogenous glucose production persists despite exogenous feeding, and anabolic resistance predominates. Large randomized trials have shown that early full caloric feeding does not improve outcomes and may prolong ICU stay or mechanical ventilation. Permissive underfeeding (approximately 12 - 25kcal/kg/day) during this phase appears safe and appropriate.^(2–4)^

As inflammation decreases and metabolic stability improves, energy requirements increase. During recovery and weaning, caloric provision should progressively approach resting energy expenditure (REE). Indirect calorimetry remains the most accurate method for estimating REE, particularly in patients with prolonged ICU stays or difficult weaning. However, targeting full measured REE too early has not shortened ventilation duration.^(5,6)^

A gradual, phase-adapted escalation of energy delivery appears more consistent with current evidence than rapid achievement of calculated targets.^(5)^

### Protein provision: moderation over enthusiasm

Skeletal and respiratory muscle wasting begins early in critical illness. Although increasing protein intake seems intuitively protective, recent high-quality trials (EFFORT-Protein, PRECISe, EAT-ICU) have not demonstrated benefit from high protein delivery (≥ 2.0 - 2.2g/kg/day), and possible harm has been reported in selected subgroups.^(7–9)^ A recent meta-analysis suggested a potential association between higher protein intake and increased mortality.^(10)^ These data support a moderate strategy:

– Acute phase: 0.8 - 1.2g/kg/day
– Recovery/weaning phase: cautious increase based on tolerance and clinical evolution

Muscle preservation alone does not guarantee successful weaning. Diaphragm function, neuromuscular coordination, sedation exposure, and early mobilization are equally relevant determinants. Protein should therefore be integrated into a broader recovery framework rather than considered a single therapeutic lever (Table 1).

**Table 1:** Phase-adapted nutritional strategy during liberation from mechanical ventilation

Phase of critical illness	Metabolic characteristics	Energy strategy	Protein strategy	Key monitoring parameters
Acute catabolic phase	High inflammation, insulin resistance, and persistent endogenous glucose production	Permissive underfeeding (≈ 12 - 25kcal/kg/day)*	0.8 - 1.2g/kg/day	Glycemia, insulin requirements
Transition phase	Decreasing inflammation, partial anabolic recovery	Gradual alignment toward measured REE†	Progressive increase if tolerated	Indirect calorimetry, phosphate levels
Weaning/recovery phase	Increased diaphragm workload, active rehabilitation	Target measured REE; avoid overfeeding and excess carbohydrate load	Individualized escalation according to clinical stability	Diaphragm ultrasound, CO_2_ production, functional status

REE - resting energy expenditure.

* Permissive underfeeding refers to hypocaloric feeding during early acute critical illness and does not imply protein restriction below recommended targets; † measured resting energy expenditure is preferably assessed by indirect calorimetry; predictive equations may be inaccurate in prolonged critical illness.

### Metabolic tolerance and the risk of overfeeding

Metabolic tolerance is central during the weaning phase. In acute illness, insulin resistance is common, and endogenous glucose production is incompletely suppressed. Excess caloric intake, particularly carbohydrates, may increase carbon dioxide production, potentially adding ventilatory burden in patients with limited respiratory reserve, particularly in conditions such as chronic obstructive pulmonary disease (COPD).^(1)^

Insulin requirements provide a pragmatic surrogate marker of metabolic tolerance. Persistently elevated insulin needs may indicate that nutrient delivery exceeds metabolic capacity and should prompt reassessment of caloric targets.

Circulating visceral proteins such as albumin, prealbumin, transferrin, and retinol-binding protein are strongly influenced by inflammation, capillary leakage, and hepatic reprioritization during the acute-phase response.^(1)^ Their concentrations correlate poorly with protein intake or short-term anabolic response and should not be used to titrate nutritional therapy during weaning. Even nitrogen balance calculations and insulin-like growth factor (IGF-1) levels provide limited insight in the presence of ongoing catabolism and systemic inflammation.

High-fat, low-carbohydrate regimens have been proposed to reduce carbon dioxide (CO_2_) production, but randomized studies have shown limited impact on clinically meaningful weaning outcomes.^(1)^ Nutritional prescriptions should therefore remain dynamic and responsive to metabolic signals rather than anchored to fixed formulas.^(1)^

### Respiratory muscle function and nutritional integration

Diaphragm dysfunction is a major contributor to weaning failure. Mechanical ventilation, systemic inflammation, corticosteroid exposure, and malnutrition accelerate respiratory muscle atrophy.^(11)^ Bedside diaphragm ultrasound enables assessment of diaphragm thickness and thickening fraction, with thresholds around 30 - 35% associated with higher likelihood of successful weaning.^(12)^ In addition to ultrasound assessment, maximal inspiratory pressure (MIP) can be used as a bedside measure of inspiratory muscle strength and may be particularly useful to monitor changes over time, especially when inspiratory muscle training is implemented.^(11)^ Although not sufficient as a standalone predictor, serial assessment may identify progressive dysfunction and guide integration of nutritional reassessment with rehabilitation strategies.

**Figure 1:**
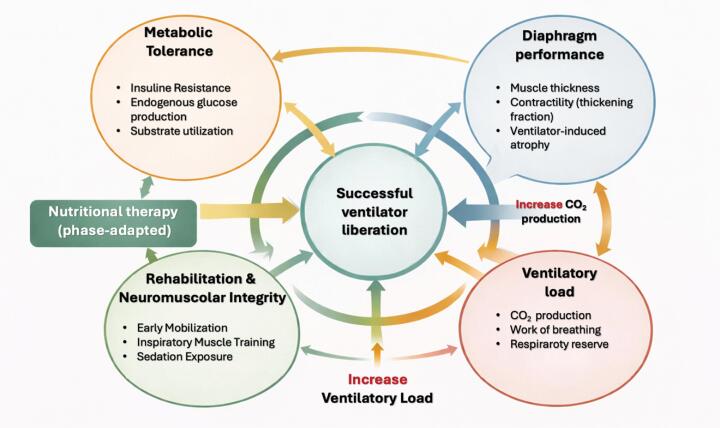
Multidimensional determinants of successful ventilator liberation.

Nutritional therapy alone cannot reverse diaphragm dysfunction. However, inadequate intake may exacerbate muscle wasting, whereas appropriately timed and individualized support, combined with early mobilization and inspiratory muscle training, may contribute to functional recovery. The interaction between metabolic tolerance, diaphragm performance, ventilatory load, and rehabilitation intensity is summarized in figure 1.

Successful liberation from mechanical ventilation depends on the interaction between metabolic tolerance, diaphragm performance, ventilatory load, and rehabilitation intensity. Phase-adapted nutritional therapy does not act directly on weaning outcomes but modulates intermediate physiological domains, including substrate utilization, carbon dioxide production, and respiratory muscle function. Optimal outcomes require coordinated alignment of metabolic and functional recovery.

### Vitamin D and micronutrients

Vitamin D deficiency is highly prevalent in critically ill patients and associated with muscle weakness and immune dysregulation.^(13)^ Nevertheless, large randomized trials such as VITdAL-ICU and VIOLET did not demonstrate a mortality benefit from high-dose supplementation.^(14,15)^ A balanced approach is warranted: assess vitamin D status, correct confirmed deficiency, and avoid indiscriminate high-dose administration. Other supplements, including creatine, branched-chain amino acids, and β-hydroxy-β-methylbutyrate, remain investigational and cannot currently be recommended in routine practice. Correction of electrolyte disturbances is also essential during weaning, particularly hypophosphatemia, which may impair diaphragmatic contractility and reduce respiratory muscle performance.^(1)^

### Integration with rehabilitation and future perspectives

Liberation from mechanical ventilation is fundamentally a functional milestone. Nutritional therapy should therefore be integrated with early mobilization, inspiratory muscle training, and structured rehabilitation.^(2)^ Future approaches should move beyond fixed caloric and protein targets and incorporate metabolic phenotyping, including inflammatory burden, insulin resistance, and anabolic responsiveness. Despite the recognized importance of nutrition, no randomized trial has specifically evaluated nutritional strategies with time to successful weaning as a primary endpoint. Such trials are needed to clarify the true clinical impact of nutritional therapy during this critical transition. The potential role of anabolic or anti-catabolic agents (e.g., selective androgen receptor modulators, β-hydroxy-β-methylbutyrate, or other pharmacologic strategies targeting muscle preservation) remains an area of active investigation. However, current evidence is insufficient to support their routine use in critically ill patients undergoing ventilator weaning.

## CONCLUSION

Optimal nutritional support is an essential component of liberation from mechanical ventilation. Current evidence favors a conservative, individualized strategy: permissive underfeeding during acute inflammation, moderate protein provision, close monitoring of metabolic tolerance, and correction of confirmed micronutrient deficiencies.

Nutritional therapy should not be viewed as an adjunctive afterthought, but as a physiologically integrated element of the weaning strategy. When aligned with the metabolic phase and combined with rehabilitation, it may become a decisive factor in successful liberation from mechanical ventilation.

Nutrition does not replace resolution of the underlying disease, but it may determine whether recovery is physiologically sustainable.

## Data Availability

Data cannot be made publicly available. This article is a mini-review and does not contain original research data, datasets, or software codes.
